# *In vitro* Model Systems for Studies Into Retinal Neuroprotection

**DOI:** 10.3389/fnins.2022.938089

**Published:** 2022-07-07

**Authors:** Yu Zhu, Bowen Cao, Arianna Tolone, Jie Yan, Gustav Christensen, Blanca Arango-Gonzalez, Marius Ueffing, François Paquet-Durand

**Affiliations:** ^1^Cell Death Mechanisms Group, Institute for Ophthalmic Research, University of Tübingen, Tübingen, Germany; ^2^Graduate Training Centre of Neuroscience, University of Tübingen, Tübingen, Germany; ^3^Molecular Biology of Retinal Degenerations, Institute for Ophthalmic Research, University of Tübingen, Tübingen, Germany

**Keywords:** retinitis pigmentosa (RP), protein kinase G (PKG), neurodegeneration, toxicity testing, drug development

## Abstract

Therapy development for neurodegenerative diseases of the retina constitutes a major unmet medical need, and this may be particularly relevant for inherited diseases of the retina, which are largely untreatable to this day. Therapy development necessitates appropriate models to improve the understanding of the underlying degenerative mechanisms, as well as for the testing and evaluation of novel treatment approaches. This review provides an overview of various *in vitro* model systems used to study retinal neuroprotection. The *in vitro* methods and technologies discussed range from primary retinal cell cultures and cell lines, to retinal organoids and organotypic retinal explants, to the cultivation of whole eyeballs. The advantages and disadvantages of these methods are compared and evaluated, also in view of the 3R principles (i.e., the refinement, reduction, and replacement of live animal testing), to identify suitable *in vitro* alternatives for *in vivo* experimentation. The article further expands on the use of *in vitro* models to test and evaluate neuroprotective treatments and to aid the development of retinal drug delivery systems. Among the pharmacological agents tested and characterized *in vitro* are such that interfere with aberrant cyclic guanosine monophosphate (cGMP) -signaling or such that inhibit the activities of poly (ADP-ribose) polymerase (PARP), histone deacetylases (HDAC), calpain-type proteases, as well as unfolded protein response-related stress. We then introduce nanoparticle-based drug delivery systems and discuss how different *in vitro* systems may be used to assess their efficacy in the treatment of retinal diseases. The summary provides a brief comparison of available *in vitro* models and relates their advantages and limitations to the various experimental requirements, for instance, for studies into disease mechanisms, novel treatments, or retinal toxicity. In many cases, combinations of different *in vitro* models may be required to obtain a comprehensive view of the efficacy of a given retinal neuroprotection approach.

## Introduction

The retina is a light-sensitive neuronal tissue located at the posterior part of the eyeball. It is arranged in three layers of cells, namely the outer nuclear layer (ONL), the inner nuclear layer (INL), and the retinal ganglion cell (RGC) layer. The ONL harbors photoreceptors, which are unique neurons dedicated to converting light into electrochemical signals and as such essential for vision. Two different types of photoreceptors are distinguished: Rod photoreceptors respond to dim light and enable vision at night, whereas cone photoreceptors respond to bright daylight and mediate high-resolution and color vision (Kolb et al., 2001).

The retina may suffer from a variety of neurodegenerative diseases, including common diseases such as glaucoma or age-related macular degeneration (AMD) and rare, inherited retinal degeneration (IRD). While glaucoma affects RGCs that form the optic nerve and relay visual information to the brain, AMD and IRD affect photoreceptors. Accordingly, IRD is typically caused by mutations in genes expressed in rod photoreceptors (Berger et al., 2010). However, rod degeneration is usually followed by a secondary loss of cones, leading to a characteristic two-stage disease progression, in which first night vision and then high-acuity daylight vision is lost (Kennan et al., 2005; Guadagni et al., 2015). In AMD, on the other hand, the disease phenotype appears to be linked primarily to a loss of cone photoreceptors, even though rods are also affected by the disease (Curcio et al., 2000).

To this day, neurodegenerative diseases of the retina remain poorly treatable, especially when it comes to diseases affecting photoreceptors, which are essentially untreatable (Power et al., 2020a). This situation creates a strong need for models that can accurately reproduce disease pathogenesis and allow the testing of new treatments for retinal neuroprotection. For the purposes of this review, we will discuss *in vitro* models for retinal diseases, focusing especially on models for photoreceptor diseases.

## Part 1: Overview of *In Vitro* Model Systems for Retinal Diseases

### Cell Culture-Based Systems as *in vitro* Models for Retinal Degenerative Diseases

Cell cultures are in many ways ideal for scientific studies: They are relatively easy to handle, inexpensive, and relatively homogenous in their cellular composition. Most cell lines can be propagated almost indefinitely, although, with increasing passage number, changes in culture properties and composition can occur and should be considered. In the context of IRD research, cell cultures can be used, for instance, for initial drug screenings before translating into *in vivo* experiments (Vighi et al., 2018). Often such studies involve the overexpression of mutant IRD genes critical to retinal function as a way to understand the pathological mechanisms induced by these mutations (McKeone et al., 2014). Among these, mutations in the rhodopsin (*RHO*) gene may be the most studied and are classified into more than seven classes based on their subcellular properties and biochemical characteristics (The human gene mutations database^1^; information retrieved in June 2022). This section describes the application of different cellular models for research into retinal disease pathologies, focusing on IRD and photoreceptor-related diseases.

#### Cell Cultures Derived From Immortalized Cell Lines

##### Photoreceptor-Like Cells

###### The 661W Cell Line

661W cells are an immortalized mouse cell line derived from transgenic mice expressing the SV40 T antigen under the control of the human inter-photoreceptor retinol-binding protein promoter (Tan et al., 2004). They express a number of cone photoreceptor markers, such as blue and green cone opsin, transducin, and cone-arrestin, yet at much lower levels than mature photoreceptors. 661W cells also display a neuronal shape and protrusion formation, showing some morphological resemblance with photoreceptors (Tan et al., 2004). They have been widely used to study cone degeneration (Mencl et al., 2018). One recent study also showed that 661W cells might be used for studying retinal ciliopathies as these cells grow long primary cilia similar in structure to those in cone photoreceptor outer segments and localize many cilium proteins to the axoneme, membrane, and transition zone (Wheway et al., 2019). On the other hand, 661W cells also express rod photoreceptor and bipolar cell markers (Mencl et al., 2018). This cell line was successfully used for studies of retinitis pigmentosa (RP) caused by rhodopsin mutations (Surgucheva et al., 2005). However, 661W cells have specific limitations, for example, relatively a low transfection efficiency of around 10% (Griciuc, 2010) and a low protein expression level for photoreceptor-specific proteins (Wheway et al., 2019).

Remarkably, a recent study reported the generation of a new clone derived from 661W cells, named 661W-A11, which stably overexpresses the neural retina leucine zipper (NRL) transcription factor, driving cell differentiation toward the rod photoreceptor fate (Huang et al., 2021). Furthermore, compared with 661W cells, 661W-A11 cell lines showed a significant increase in the expression of rod-specific genes but not of cone-specific genes, which makes this new cell line potentially suitable as a cell culture model to study IRD and the development of high-throughput *in vitro* drug screening systems. Altogether, the 661W cell line may mimic certain characteristics of photoreceptors, allowing for exploration of specific aspects of retinal disease mechanisms, yet it is still far from representing primary photoreceptors.

###### The Y-79 Retinoblastoma Cell Line

The Y-79 cell line was originally established from a human retinoblastoma tumor and is thought to be derived from primitive multipotential retinoblasts (Reid et al., 1974; Kyritsis et al., 1984). Like 661W cells, the Y-79 line has also been used as a model for photoreceptors. Y-79 cells are usually maintained in suspension culture in an undifferentiated state, while some cells can be stimulated to express characteristics of differentiated, mature retinal cell types (Kyritsis et al., 1985). Y-79 cells express a comparable mRNA level of rod *PDE6A* and cone *PDE6C* genes while the transcript encoding the rod *PDE6B* subunit was 10 times more abundant. This made the Y-79 line be a good model for studying transcriptional regulation of rod-specific genes (Di Polo and Farber, 1995). However, later studies reported that Y-79 cells were not able to express functionally active cone PDE6A protein and suggested that the expression of a fully active stable PDE6 enzyme required other post-transcriptional events that did not occur or were inhibited in Y-79 cells (White et al., 2004). Moreover, this cell line appears to be difficult to transfect and lacks reproducibility of gene transfer, reducing the utility of these cells for scientific research (White et al., 2001).

###### Reprogramming Cells Into Photoreceptor-Like Cells

Since the generation of sensory neurons such as photoreceptors remains a challenge, reprogramming other cell types into photoreceptor-like cells has been developed as an alternative method. Two major approaches have been considered, gene-induced and chemical (or pharmacological) reprogramming. An example of gene-induced reprogramming is the introduction of human NEUROD (*NRD*) or *NGN1* genes to hTERT RPE-1 (human telomerase-immortalized retinal pigmented epithelial) and ARPE-19 cell cultures, respectively, or the transfection of *NGN1* or *NGN3* genes into primary retinal pigment epithelial (RPE) cell cultures (from mouse or porcine origin), changing gene expression and cellular morphologies, suggesting that they are responsive to the reprogramming (Yan et al., 2013).

Furthermore, chemical reprogramming of fibroblasts by administering a set of small molecules [valproic acid (V), CHIR99021 (a GSK3 inhibitor; C), RepSox (R), and forskolin (F), together denoted VCRF] has been shown to induce the transformation of fibroblasts into rod photoreceptor-like cells based on gene expression profiling (Mahato et al., 2020).

Besides that, single colonies derived from the pigmented ciliary margin cells of adult mouse eyes have been generated. They can clonally proliferate *in vitro* to form spheroid colonies of cells that can differentiate into retinal-specific cell types, including rod photoreceptors, bipolar neurons, and Müller glial cells (MGCs) (Tropepe et al., 2000). This method has been applied in IRD research, using retinoic acid to drive the differentiation into photoreceptor cells (Sanges et al., 2006). While reprogramming somatic cells with stem cell-like behavior remains a possibility for retinal research, the more recent discovery of induced pluripotent stem cells has opened further and potentially more interesting possibilities (see organoid cultures below).

##### Other Cell Lines

###### SK-N-SH Cells

SK-N-SH is a human neuroblastoma cell line developed in the early 1970s (Biedler et al., 1973) that expresses multiple neurochemical markers and exhibits a neuronal phenotype (Biedler et al., 1978). Furthermore, SK-N-SH cells respond to numerous insults, including β-amyloid overexpression, mitochondrial permeability transition, and serum deprivation, indicating that this cell line may be useful in the assessment of neurotoxicity and neuroprotection (Ba et al., 2003). Accordingly, SK-N-SH cells have been used as a model for identifying and characterizing modulators of mutant rhodopsin processing and aggregation (Chapple and Cheetham, 2003; Mendes and Cheetham, 2008; Griciuc et al., 2010; Athanasiou et al., 2017). Besides, this cell line was suggested as an *in vitro* model for studying neuroprotection mechanisms in retinal degenerative diseases (Watters and Dorsa, 1998; Green et al., 2001; Wen et al., 2004; Wang et al., 2006). Limitations of the SK-N-SH cell line include low transfection efficiencies of around 15% (Griciuc, 2010) and low expression of retina-specific genes and proteins.

###### HEK293 and COS Cells

HEK293 cells and COS cells are common *in vitro* models that have been widely used in cell biology and biotechnology research for many years. The HEK293 immortalized cell line was derived from human embryonic kidney cells from a female fetus (Graham et al., 1977; Graham, 1992; Kavsan et al., 2011). They exhibit high transfection efficiency and can produce proteins most similar to those naturally synthesized in humans (Dumont et al., 2016). Several further cell lines have been derived from HEK293 cells, including HEK293T (Lin et al., 2014), HEK293E (Kim et al., 2009b), HEK293H (Bloom et al., 2001), and HEK293S (Lin et al., 2014). Among these, HEK293T and HEK293S appear to be the most commonly used hosts.

COS cells are fibroblast-like cell lines derived from the kidney tissue of the African green monkey (*Chlorocebus aethiops*), obtained by immortalizing CV-1 cells (Jensen et al., 1964) by transformation with an origin defective mutant of SV40 virus that can produce large T antigen (Gluzman, 1981). Three COS lines were created (COS-1, COS-3, and COS-7), of which two are commonly used (COS-1 and COS-7).

While HEK293 and COS cells are non-neuronal in origin, they have been used extensively in neurodegeneration research. For instance, in neurodegenerative diseases, such as Parkinson’s, Alzheimer’s, and Huntington’s disease, HEK293 and COS cells are often used together to characterize the pathology and define protein accumulation (Hering et al., 2004; Lammich et al., 2004; So et al., 2013; Magno et al., 2019). Likewise, in IRD-research causative mutations often lead to protein conformational defects, for instance, in the *RHO* or *PDE6* genes. Such mutants can be easily overexpressed in HEK293 and COS7 cells for experimental investigation. Consequently, numerous studies based on these two cell lines examined protein behavior in IRD (Mendes and Cheetham, 2008; Griciuc et al., 2010; Gopalakrishna et al., 2016).

###### MIO-M1 Cells

The MIO-M1 cell line (Moorfields/Institute of Ophthalmology-Müller 1) was the first immortalized human MGCs line derived from a female corneal donor (Limb et al., 2002b). Cell cultures derived from the MIO-M1 line are often characterized as MGCs, based on the expression of specific makers and morphological analysis. However, the effect of immortalization, high passage number, and expression on non-MGC markers have received little attention. Indeed MIO-M1 cells have been shown to express neural stem cell markers such as tubulin, SOX2, PAX6, CHX10, and NOTCH1, when exposed to various extracellular matrix and growth factors *in vitro* (Lawrence et al., 2007). Other investigations found MIO-M1 to express features of mature cells, producing hepatocyte growth factor and vascular endothelial growth factor (Hollborn et al., 2005) and matrix metalloproteinases 1, 2, and 9 (Limb et al., 2002a; Limb et al., 2005). Altogether, these findings suggest that MIO-M1 cells are not truly representative of MGCs, limiting the usefulness of this cell line for investigations on MGC physiology.

###### Microglial Cell Lines

The role of microglial cells in retinal degenerative diseases appears ambiguous, but the general consensus seems to be that these cells are mostly involved in secondary pathologies rather than the primary changes causing IRD (Sancho-Pelluz et al., 2008; Ferrer-Martin et al., 2015; Funatsu et al., 2022). Nevertheless, the relatively recently introduced BV2 and MG5 microglial cell lines may provide new attractive opportunities for studying microglial properties and the possible roles of these cells in photoreceptor degeneration (Chumsakul et al., 2020; Ozaki et al., 2022).

#### Primary Cells

For decades, researchers have attempted to isolate different retinal cell types from a variety of different species, such as fish, amphibians, rodents, primates, and humans (Han et al., 2000). The physical separation of retinal cells from each other typically requires enzymatic and mechanical intervention. After dissociation, cell types may be identified by their morphology, specific markers, or characteristic measurements, such as the transepithelial resistance, to assess RPE cell confluence and cell density (Skaper, 2012). The primary cell cultures mentioned in this review include rod and cone photoreceptors, MGCs, and RPE cells.

##### Photoreceptor Cells

The culture of primary photoreceptor cells has been challenging for many years. While various protocols and methods have been tried (Han et al., 2000), including protocols used for MGC culture (see below), the viability of isolated photoreceptors is typically limited to 1–2 days. Early in 1972, protocols for isolating photoreceptors from turtles were already available (Lam, 1972). This was followed by the addition of neurotrophic factors (Skaper, 2012; Forouzanfar et al., 2020) to the cultures, such as the ciliary neurotrophic factor (CNTF) and brain-derived neurotrophic factor (BDNF), to promote the structural integrity and survival of photoreceptor cells. Isolated cone photoreceptor cultures obtained from rat retina based on a peanut agglutinin (PNA) lectin–panning procedure (Skaper, 2012) show viability typically limited to 1–2 days. Hence, there are significant challenges when trying to obtain adequate photoreceptor cultures, including the short-time survival after isolation and the loss of photoreceptor-specific structures and compartments (i.e., inner and outer segments and ribbon synapse) (Yang et al., 2001).

##### Müller Glia Cells

Müller glial cells form a network that connects other retinal cells, extending vertically through the entire retina, from the RGCs to photoreceptors. MGCs play a crucial role in metabolism, support, and modulation of neuronal excitability by releasing and transporting neurotransmitters (Eastlake et al., 2021). They may also show progenitor cell characteristics in the adult retina (Lawrence et al., 2007). Moreover, MGCs can respond to retinal injury by secreting neuroprotective factors reducing rod photoreceptor cell death in IRD mouse models (Del Rio et al., 2011; Roche et al., 2016).

Primary MGCs are used relatively often cultivated after enzymatic and mechanical isolation being relatively similar across different species (Hicks and Courtois, 1990; Han et al., 2000; Liu et al., 2017). MGC isolation after dissociation of primary retinal cells is facilitated greatly by the fact that MGCs are essentially the only cell type that survives for more than 2 days in culture, while all other neuronal cell types (e.g., photoreceptors, see above) rapidly die off. However, after 1–2 days in culture, primary MGCs rapidly dedifferentiate and assume a fibroblast-like phenotype (Hauck et al., 2003).

##### Retinal Pigment Epithelial Cells

The RPE consists of pigmented cells essential for photoreceptor cell survival. They are highly polarized cells that mediate the recycling of the photopigment retinal, phagocytose the rod- and cone- outer segments (Schnichels et al., 2021), and protect photoreceptors against photooxidation (Strauss, 2005). RPE cells are linked by tight junctions and form a monolayer that constitutes a part of the outer blood-retinal barrier (BRB), which regulates the movement of solutes and nutrients from the choroid to the sub-retinal space (Campbell and Humphries, 2012). Moreover, they are immunocompetent (at least in part) and provide the molecular and cellular interphase between the neuroretina and the choriocapillaris (Armento et al., 2021b). Immortalized RPE cells, such as the RPE-1 cell line, have been widely used for experimental purposes, especially in AMD research, as they degenerate in this disease (Armento et al., 2021a).

The RPE is located at the back of the eyeball, making *in vivo* studies difficult. However, RPE cells lend themselves to *in vitro* studies since they typically preserve their complete functional phenotype and cell contacts (Oswald and Baranov, 2018). The first human RPE culture goes back to 1973 (Mannagh et al., 1973), and since then, many strategies for RPE culturing have been developed (Skaper, 2012). Common problems of RPE cultures include pigmentation loss, low cell-substrate adhesion, alterations in cell morphology, and low long-term viability of cells (Blenkinsop et al., 2013). In part to overcome these issues, different cell lines have been developed that can mimic most of the RPEs properties.

###### ARPE-19 and hTERT RPE-1 Cells

The ARPE-19 cell line was developed from the RPE cell layer of a human eye post-mortem (Kozlowski, 2015). This cell line displays many of the properties of native RPE cells; for example, the cobblestone morphology, which is characteristic of RPE cells (Dunn et al., 1996), the formation of polarized structures on porous filter supports (Dunn et al., 1996), the expression of cellular retinaldehyde-binding protein (CRALBP), one of the RPE-selective markers (Ablonczy and Crosson, 2007). Because of these RPE-like properties, the ARPE-19 cell line has frequently been used in the research of retinal disorders, including AMD (Kozlowski, 2015), RP (Hulleman et al., 2016), retinal ciliopathies, and Leber’s congenital amaurosis (van Wijk et al., 2009). Similarly, another cell strain, hTERT RPE-1, an hTERT immortalized female RPE cell line, is also routinely used in molecular biology studies of retinal ciliopathies (Adams et al., 2007; Spalluto et al., 2013). The findings suggest that hTERT RPE-1 cells could serve as a model system for studying the molecular pathways, including reciliation in the late G1 of the cell cycle and those that stimulate cilium disassembly.

### Retinal Organoid Cultures

The ground-breaking work of Yamanaka and colleagues (Takahashi and Yamanaka, 2006) made it possible to generate induced pluripotent stem cells (iPSC) from somatic cells. iPSCs can generate most cell types and tissues of an organism. This has given rise to numerous applications, including the generation of human stem cell-derived retinal RPE cells and retinal organoids. Concerning the retina, iPSCs can differentiate into RPE cells and cells of the neural retina, including rods and cones. The process of harvesting somatic cells and reprogramming them into virus-free human iPSCs (hiPSC) and from there to retinal organoids has been standardized in various laboratories (Liebau et al., 2019; Cobb et al., 2021).

A variety of signaling pathway modulators promote differentiation to retinal cells (e.g., the TGFß, Wnt, Nodal, and BMP signaling) in a defined time frame that takes up to 52 weeks to complete (Achberger et al., 2019b). Furthermore, animal experiments and even clinical trials have been initiated with the transplantation of stem cell-derived retinal cells into the diseased or degenerated retina (Mandai et al., 2017). As such, hiPSC-derived organoids can serve as a human model system to study cell development, function, and disease mechanisms but also represent a new source of individual cell material for future cell-based therapies. Moreover, the hiPSC-derived retinal organoids obtained from individual donors suffering, for instance, from IRD, can provide retina-like structures mimicking the disease. Such organoids feature not only the genetic set-up of the donor but also the retinal context, including retinal anatomy and physiology, as well as light reception.

Three-dimensional organoids derived from hiPSC resemble rudimentary optic vesicle-like structures with a retinal layering similar to *in vivo* conditions (Nakano et al., 2012; Zhong et al., 2014). The resulting organoids contain the most relevant retinal cell types in a physiological layering (Achberger et al., 2019a). As such, three-dimensional retinal organoids from hiPSC cells comprise all main cell types of the retina in a layered manner, such as photoreceptors, amacrine, horizontal, bipolar cells, as well as RGCs and MGCs (Canto-Soler et al., 2016). An exception is the RPE, which forms amorphous structures apart from the neuroretina. While attempts have been made to co-culture retinal organoids with RPE, these have so far had only limited success (Achberger et al., 2019b). Still, immunofluorescence shows the outer and inner limiting membrane and synaptic connections between the neuronal layers (Achberger et al., 2019a).

Nevertheless, retinal organoids are still facing a variety of limitations: They lack functional maturation of differentiated cells, especially with respect to mature photoreceptors, which do not grow proper outer segments and do not connect well with the RPE. Microglial cells are missing in retinal organoids, and RGCs do not reach full maturation and are unable to bundle to an optic nerve-like structure or connect to the brain. The inner limiting membrane produced in an interplay between RGCs and MGCs end-feet lacks the Bruch’s membrane, separating the retina from the choroid and choriocapillaris. Also, the inner retinal vasculature is lacking. As such, and due to the lack of physiological perfusion from the bloodstream, the delivery of nutrients, metabolites, and oxygen remains entirely artificial.

### Organotypic Retinal Explants

Cell lines and primary cell cultures are suitable for studying intrinsic cell characteristics but do not allow for the investigation of complex interactions between different cell types arranged in specific structures and organizational units. Yet, the retina is a complex network governed by a multitude of cell-to-cell interactions, both under physiological and pathological conditions. Here, organotypic retinal explant cultures allow long-term studies on a retina that has conserved its normal histotypic context and that can be easily intervened for experimental purposes (Caffe et al., 1989; Caffé et al., 1993). Over the last decades, several different methods have been used. For instance, the culture of complete mouse eyecups allowed to maintain retinal structures and photoreceptor viability for around 6 days in culture (Müller et al., 2017). The explanted retina cultured without RPE, displayed characteristic morphological alterations and extensive photoreceptor cell death starting between 2 to 4 days *in vitro* (Ferrer-Martín et al., 2014; Müller, 2019). Nevertheless, retinal explants cultured without RPE but with the vitreous remaining attached to the inner limiting membrane have been used for short-term (48 h) studies into drug delivery through the vitreoretinal interface (Tavakoli et al., 2020). In addition, the whole retinal vasculature degenerates rapidly in the absence of blood supply.

The approaches mentioned above were suitable only for investigations limited to *in vitro* duration of a few days. For longer-term studies, organotypic retinal explants with intact RPE were developed in the late 1980s (Caffe et al., 1989). The preparation of retinal explants (Belhadj et al., 2020) includes the separation of the retina from choroid and sclera, the removal of lenses, iris, ciliary body, vitreous, and optic nerve, and the cutting of the retinal cup into a four-leaf clover shape to flatten the retina on the culturing membrane. The cultivation of the retina in defined R16 medium, free of serum and antibiotics, allows culture periods of at least 4 weeks. An important aspect of such retinal explants is that they must be cultured on a suitable porous membrane in an air-liquid interface, such that the retina is covered by only a thin film of liquid created by the surface tension of water. This is critical to ensure sufficient oxygenation of the retina. Retinal explants prepared in this way may be used for different experimental applications and technologies, such as histology, patch-clamp recording (Moritoh et al., 2010), or multi-electrode-array (MEA) recording (Reinhard et al., 2014).

While organotypic retinal explants with RPE cannot replace *in vivo* experimentation completely, there are many merits compared to *in vivo* experiments, including but not limited to: lower experimental complexity, cost-effectiveness, the ability of experimental manipulations under entirely controlled conditions, the avoidance of animal pain and suffering, and a reduction of animals needed since retinas from both eyes can be used (Belhadj et al., 2020). These advantages make retinal explants an almost ideal and indispensable system for identifying and studying new potential treatments for retinal diseases. A limitation, however, is the relatively complex explantation and dissection procedure that requires appropriate training and experience. In addition, some of the limitations mentioned for organoid cultures (see above; lack of perfusion, microglial cells, Bruch’s membrane) likewise apply to organotypic retinal explants.

### First Attempts: Whole Eye Culture

Before the event of modern cell and organ culture systems, *in vitro* studies on the retina typically employed whole eye cultures, starting in the second half of the 19th century, often using frog eyes (Kuhne and Steiner, 1880; Kühne, 1881; Brücke and Garten, 1907). A limitation in these studies was that the electroretinographic response (ERG) to a light stimulus was typically lost within 1 h from enucleation, a phenomenon attributed to tissue degeneration. However, the ERG response could be preserved for up to 24 h when isolated frog eyes were kept under a 100% oxygen atmosphere (Bauereisen et al., 1956), indicating that sufficient oxygenation was critical to retinal function. Similar studies in whole-eye cultures obtained from rabbits (Böck et al., 1964) and rats (Suga, 1972) demonstrated the importance of temperature and sufficient glucose supply for retinal function.

In an attempt to control all these parameters, in 1960, vascular perfusion of the entire mammalian eye after enucleation was first carried out (Lele and Grimes, 1960). Afterward, vascular perfusion was applied to different mammalian species (Niemeyer, 2001; Koeberle et al., 2006), including rats, rabbits, dogs, monkeys, cats, and bovines. This allowed the recording of retinal function using an ERG, light-evoked optic nerve response, and standing potential. Perfused cat eyes could be cultured for 8–10 h while still displaying light-responsiveness as assessed *via* ERG (Niemeyer, 1975). With perfusion beyond 9 h, photoreceptors swelled slightly, while RPE cells, RGCs, and other cell types in the INL showed mild and moderate loss (Koeberle et al., 2006). Perfusion times over 10 h led to retinal detachments and overall function loss (Niemeyer, 2001).

Among the advantages of using vascular perfusion of whole eyes are the possibilities to combine the monitoring of electrophysiological activity with the arterial application of an intervention without influence by extraocular regulation, such as systemic vascular changes. In addition, the structural integrity and the pathophysiological performance of the retina allowed studies on the pharmacology of vascular dynamics and studies into acute retinal toxicity (Niemeyer, 2001). Nevertheless, an inevitable disadvantage of whole-eye culture is the relatively limited time frame for experimental interventions (from minutes to several hours), which does not allow investigating longer-term effects.

## Part 2: Studying Retinal Neuroprotection *In Vitro*

### Pharmacological Agents

#### Targeting Cyclic Guanosine Monophosphate-Signaling

The mechanisms underlying retinal neurodegenerative disorders are complex and far from being completely understood. *In vitro* models, such as cell cultures and organotypic retinal explants, are widely used for IRD research as these models are suitable for studying components of the photoreceptor cell death pathway, such as cyclic guanosine monophosphate (cGMP), calpain-type proteases, histone deacetylases (HDAC) and poly (ADP-ribose) polymerase (PARP).

Increased cGMP levels have been connected to retinal photoreceptor degeneration already in the early 1970s (Farber and Lolley, 1974). Moreover, high levels of cGMP have been observed in different animal models for IRD, suggesting that excessive cGMP signaling may be a common event in several mutations causing photoreceptor degeneration (Power et al., 2020a). One way to study the mechanism of cell death triggered by abnormal cGMP accumulation is the inhibition of its two prototypic targets: Cyclic nucleotide-gated (CNG) channels and protein kinase G (PKG), both of which have been connected to photoreceptor cell death (Farber and Lolley, 1974; Paquet-Durand et al., 2009). Both photoreceptor-like cell cultures and organotypic retinal explant cultures have been employed to target CNG channels and PKG and have contributed valuable insights into the photoreceptor death pathway (Vighi et al., 2018; Tolone et al., 2021; Das et al., 2022).

Recently, PKG inhibition mediated by cGMP analogs has attracted the interest of IRD researchers as a potential neuroprotective strategy to slow down photoreceptor death (Paquet-Durand et al., 2019). cGMP analogs mimic the overall structure of cGMP while carrying substitutions in certain residues that enable them to inhibit specific cGMP-signaling targets (Schwede et al., 2000). PKG-inhibiting cGMP analogs carry a phosphorothioate configured as Rp, which allows them to counteract PKG activation (Zhao et al., 1997). Two novel cGMP analogs with strong protective abilities on degenerating photoreceptors have recently been identified using *in vitro* systems: Photoreceptor-like cell cultures allowed an initial screening of several compounds, while organotypic retinal explants cultures derived from different IRD mouse models confirmed the neuroprotective capacities of these cGMP analogs (Vighi et al., 2018; Tolone et al., 2021). Furthermore, the use of retinal explant cultures treated with inhibitory cGMP analogs also led to the identification of novel PKG targets that may be involved in the cell death mechanism of IRD (Roy et al., 2021; Roy et al., 2022).

#### Poly (ADP-ribose) Polymerase Inhibition Brings Retinal Neuroprotection

Poly (ADP-ribose) Polymerases are DNA repair enzymes (Ko and Ren, 2012), comprising a family of 17 isoforms that share a conserved catalytic domain (Krishnakumar and Kraus, 2010). The best-characterized isoform within this family is PARP-1, which catalyzes the PARylation of target proteins by consuming nicotinamide adenine dinucleotide (NAD^+^) (Houtkooper et al., 2010; Morales et al., 2014; Bai, 2015; Lord and Ashworth, 2017; Murata et al., 2019). Over-activation of PARP forces the cell to synthesize NAD^+^ using salvage pathways, which may cause subsequent ATP depletion (Houtkooper et al., 2010; Xie et al., 2020). Hence, persistent PARP activation results in a specific form of cell death termed PARthanatos (David et al., 2009).

In *in vitro* retinal cultures derived from *rd1* mice, PARP inhibition reduced the number of cells exhibiting death markers (Paquet-Durand et al., 2007; Sahaboglu et al., 2016; Sahaboglu et al., 2020; Yan et al., 2022), suggesting the neuroprotective effect of PARP inhibitors, and highlighting the importance of a novel cell death pathway that is triggered by high cGMP-levels and likely related to PARthanatos (Yan et al., 2021). The success of PARP inhibitors in *in vitro* experiments makes PARP inhibition appear as an attractive strategy for therapy development, notably because a number of already clinically approved PARP inhibitors offer accelerated and low-cost progress in disease combating. This may be of particular interest in the case of rare IRD-type diseases such as RP (Sahaboglu et al., 2020).

#### Histone Deacetylases Inhibition in Retinal Degeneration

Histone function is modulated by multiple posttranslational modifications, including reversible acetylation of the amino-terminal ε-group of lysine residues. Histone acetylation is tightly controlled by a balance between the opposing activities of histone acetyltransferases (HATs) and HDACs. There are 18 potential human HDACs grouped into four classes (Seto and Yoshida, 2014).

With the help of *in vitro* retinal cultures, IRD was reported to be associated with excessive HDAC activation, and *in vitro* HDAC inhibition strongly reduced photoreceptor cell death (Sancho-Pelluz et al., 2010; El Bahhaj et al., 2014). Since then, HDACs have been considered part of the cGMP-dependent cell death mechanism (Arango-Gonzalez et al., 2014). On the other hand, sirtuin-type HDACs (class III HDACs) are considered to be neuroprotective (Jiang et al., 2011; Gomes et al., 2018; Xu et al., 2018). Given the pleiotropic effects of HDAC activity, HDAC inhibitors, such as trichostatin A (TSA), suberoylanilide hydroxamic acid (SAHA), or valproic acid (VPA), often target multiple HDACs, and the biological consequences are often unpredictable and underappreciated (Witt et al., 2009; Falkenberg and Johnstone, 2014). Here, *in vitro* experimentation may offer a rapid and economical way of solving essential open questions for the therapeutic development of HDAC inhibitors for IRD.

#### Calpain Inhibition in Retinal Degeneration

Calpain belongs to a 15-member family of the calcium-dependent thiol proteases (Perrin and Huttenlocher, 2002; Curcio et al., 2016). The best-characterized calpains in the central nervous system are two distinct, heterodimeric subtypes: μ-calpain and m-calpain, also known as calpain-1 and calpain-2 (Cheng et al., 2018). Like HDACs, calpains may show pleiotropic effects, in which calpain-1 could be neuroprotective, while calpain-2 could be neurodegenerative (Baudry and Bi, 2016; Baudry, 2019; Wang et al., 2020). In addition, over-activated calpain has been reported in *rd1* photoreceptors *in vivo*, and calpain inhibition with the endogenous calpain inhibitor calpastatin on *in vitro rd1* retinal explant cultures attenuated photoreceptor degeneration (Paquet-Durand et al., 2006; Power et al., 2020b; Yan et al., 2022), which suggests that calpains form part of IRD cell death mechanisms. The only specific inhibitor for classical calpains currently available, calpastatin, blocks calpain-1 and calpain-2, calpain-8, calpain-9, and calpain-8/9 (Wendt et al., 2004; Hata et al., 2007; Hata et al., 2010; Ono et al., 2016). Thus, further *in vitro* studies are needed to further elucidate the diverse roles of different calpain isoforms during IRD.

#### Drugs Targeting Complex Cellular Processes

##### Small Molecules

In addition to the mechanisms and drug categories mentioned above, other pharmacological approaches targeting common events observed in IRD have been validated using *in vitro* studies. Physiological and pathophysiological processes such as proteostasis, neuroinflammatory response, and mitochondrial homeostasis are affected in IRD, generating the chance to identify multiple molecular targets for pharmacological intervention studies. In this context, a particularly interesting therapeutic strategy is to attenuate the stress induced by perturbance of proteostasis, that can arise, once proper protein folding, transport, and degradation of proteins are compromised. For instance, mutations in the *RHO* gene encoding for rhodopsin, that cause protein misfolding and aggregation, are the most common cause of autosomal dominant retinitis pigmentosa (adRP) (Sen et al., 2021c). Pharmacological targeting of proteostasis regulators has been proven neuroprotective for rhodopsin mutations. Inhibition of valosin-containing protein (VCP) through a small molecule inhibitor significantly reduced cell death in photoreceptors, restored physiological rhodopsin localization in the outer segment, and improved retinal function in organotypic retinal explants derived from *Rho^P23H^* transgenic rats and *Rho^P23H^* knock-in mice (Arango-Gonzalez et al., 2020; Sen et al., 2021a,c). As proof of concept, the delivery of VCP siRNA using reverse magnetofection in organotypic cultures of *Rho^P23H^* transgenic retinas effectively prevented photoreceptor cell death and attenuated retinal degeneration *in vitro* (Sen et al., 2021b). Other small molecules newly identified that rescued the transport of rhodopsin include the chaperon YC-001 which protected Abca4^–/–^ * Rdh8^–/–^ double-mutant mice from bright light-induced photoreceptor death (Chen et al., 2018).

##### Macromolecules

Recombinant neurotrophic factors have also been tested in organotypic cultures for photoreceptor neuroprotection. This included CNTF, BDNF, and glial cell-line derived neurotrophic factor (GDNF). CNTF is one of the most studied neuroprotective agents with acknowledged potential in treating diseases of the posterior eye segment (Itkonen et al., 2020) and has been found to promote cone survival (Dutt et al., 2010; Lipinski et al., 2011). BDNF delayed photoreceptor cell loss and increased the number of photoreceptor rows (Pinzon-Duarte et al., 2004); however, it was much more effective when combined with CNTF in *rd1* mouse retinal explants (Caffé et al., 2001; Azadi et al., 2007). GDNF is a member of the transforming growth factor β (TGFβ) family that promotes the survival, proliferation, and differentiation of neurons, RGCs, and photoreceptors (Kolomeyer and Zarbin, 2014). When combined with CNTF, GDNF effectively attenuated the secondary loss of cone photoreceptors on retinal explants derived from *RHO* knockout mice (Lipinski et al., 2011). Possibly, GDNF executes its neuroprotective effects on photoreceptors indirectly *via* MGCs (Hauck et al., 2006). Upon GDNF stimulation isolated RMGs secrete a mixture of factors that prolong the viability of photoreceptors in mouse retinas *in vitro* (Del Rio et al., 2011). Upon analyzing the MGCs conditioned medium using proteomics- and transcriptomic approaches, several macromolecules with photoreceptor protective properties have been identified (Hauck et al., 2014; von Toerne et al., 2014). Among these compounds, Osteopontin (OPN) and Cysteine-rich angiogenic inducer 61 (Cyr61) have shown protective effects on *rd1* photoreceptors *in vitro* (Del Rio et al., 2011; Kucharska et al., 2014).

### Drug Delivery System Development

To treat retinal diseases, intravitreal injections are commonly used, for instance, in the treatment of AMD (Garcia-Quintanilla et al., 2019) to get the administered drugs in close proximity to the target tissue. However, for most treatments, multiple injections are necessary, which increases the risk of complications (Shima et al., 2008). The administration of drugs combined with a drug delivery system (DDS) can be beneficial in improving the pharmacokinetic profile with the aim of reducing the injection frequency and limiting systemic side effects (Zeng et al., 1993; Zhang et al., 2010; Bochot and Fattal, 2012; Kamaleddin, 2017). In this regard, nanoparticles have gained interest since they can be engineered small enough to diffuse freely within the vitreous (Lee et al., 2017) and permeate relevant ocular barriers (Kim et al., 2009a). For systemic applications, targeting moieties on the nanoparticle surface can help the drug accumulate in a specific tissue or cell type in the body (Hu et al., 2019).

Since most DDS typically exhibit an improvement over a free drug solution *in vivo*, the challenge of evaluating the potential of DDS without using living animals is that suitable *in vitro* systems should closely emulate the *in vivo* conditions. A common strategy seems to be taking relevant excised ocular tissue from dead animals and investigating the DDS properties in this system (Eriksen et al., 2017; Tavakoli et al., 2021). An obvious source of the tissue samples is leftover eyes during meat production at slaughterhouses. For instance, bovine eyes have been used to prepare retinal explant cultures with the vitreous intact (vitreoretinal explant cultures) to investigate the diffusion of nanoparticles through the vitreoretinal interface (Peynshaert et al., 2017; Tavakoli et al., 2020). Here, it was found that liposomes surface-grafted with poly (ethylene glycol) polymers need to be less than 100 nm in diameter to diffuse into the retina. When the vitreous is conserved during the explantation procedure, the explant cultures are more restrictive to the nanoparticle’s ability to diffuse into the retina (Peynshaert et al., 2017). Still, it is not certain how active the cells are in such explant cultures. If active cell uptake is important for the application, retinal explant cultures derived right after animal sacrifice might be more suitable. For mouse-derived retinas, the culture can be kept viable for up to 4 weeks (see above). The long-term viability of the retina also allows for the implementation of appropriate nanoparticle cytotoxicity studies (Prajapati et al., 2021).

To analyze topical applications, the permeation of DDS through the cornea and mucus barrier has been investigated using Franz-diffusion cells loaded with appropriate *ex vivo* tissues (Bao et al., 2018; Gomez-Segura et al., 2020).

For studies into the pharmacokinetic profile of drugs in DDS, a 2-chamber flow-based system called PK-Eye™ has been developed to estimate the anterior elimination half-life of intravitreally administered drugs entirely free from biological tissue (Awwad et al., 2017). Evidence suggests that predictions on the elimination of small lipophilic molecules are less accurate, e.g., the half-life of triamcinolone acetonide was found to be 26–28 days in the model, while this was shown to be considerably faster in humans (15.4–18.6 days) due to the elimination route through the retinal-choroid-sclera pathway, which the model does not take into account (Awwad et al., 2015). The half-life of proteins like ranibizumab, on the other hand, was more accurately represented and found to be about 8 days, which is in the same range as in humans (7–9 days) (Awwad et al., 2015). Nanoparticles are primarily cleared through the anterior route (Del Amo et al., 2017), suggesting that the PK-Eye™ model might be useful for estimating their intraocular elimination half-life.

In summary, multiple *in vitro* settings have been employed in the literatures, depending on which specific property of the DDS is being investigated (e.g., cell uptake, pharmacokinetics, permeation through ocular tissue barriers). The information gained from such *in vitro* models can give useful insight into the DDS behavior and predict whether they might yield successful *in vivo* results.

## Conclusion

A large variety of very diverse *in vitro* systems is available to model human retinal diseases. Each of these systems comes with its own set of benefits and limitations, and in practice, it will be important to precisely define the experimental questions at hand to choose the appropriate *in vitro* model then. In Table 1 we have summarized the main *in vitro* systems currently available for research into retinal degeneration and neuroprotection.

**TABLE 1:** Overview of *in vitro* test systems available for retinal neuroprotection research.

*In vitro* system		Cell type (s)	Applications	References
Cell lines	661W	Cone photoreceptor; rod photoreceptor	Pharmacological research; immunohistochemistry; modeling of IRD: RP, cone degeneration, macular degeneration, retinal ciliopathies.	Tan et al., 2004; Surgucheva et al., 2005; Mencl et al., 2018; Wheway et al., 2019; Huang et al., 2021
	Y-79	Rod photoreceptor	Pharmacological research; immunohistochemistry; modeling of RP.	Kyritsis et al., 1985; Di Polo and Farber, 1995; White et al., 2004
	Reprogramming of primary cells	Photoreceptor-like cells; bipolar cells; MGCs	Pharmacological research; immunohistochemistry; modeling of RP.	Tropepe et al., 2000; Sanges et al., 2006; Yan et al., 2013; Mahato et al., 2020
	Other cell lines: SK-N-SH cells; HEK293 cells; COS cells; ARPE-19 cells; hTERT RPE-1 cells; MIO-M1 cells; BV2; MG5.	Photoreceptor-like cells; RPE cells; MGCs, stem cells? microglial cells.	Pharmacological research; immunohistochemistry; modeling of RP, macular degeneration, retinal ciliopathies, Leber’s congenital amaurosis, cone-dystrophy, cone-rod dystrophy.	Limb et al., 2002b; Chapple and Cheetham, 2003; Adams et al., 2007; Lawrence et al., 2007; Mendes and Cheetham, 2008; van Wijk et al., 2009; Griciuc et al., 2010; Spalluto et al., 2013; Kozlowski, 2015; Gopalakrishna et al., 2016; Hulleman et al., 2016; Athanasiou et al., 2017; Chumsakul et al., 2020; Ozaki et al., 2022
Primary cells	Photoreceptor cells	Photoreceptor cells	Pharmacological research; immunohistochemistry; modeling of RP.	Lam, 1972; Han et al., 2000; Skaper, 2012
	MGCs	MGCs	Pharmacological research; immunohistochemistry; modeling of IRD.	Hicks and Courtois, 1990; Hollborn et al., 2005; Del Rio et al., 2011; Liu et al., 2017
	RPE cells	RPE cells	Pharmacological research; immunohistochemistry; modeling of RP.	Mannagh et al., 1973; Oswald and Baranov, 2018
Retinal organoid cultures		All retinal cells	Pharmacological research; immunohistochemistry; human model for IRD; transplantation.	Takahashi and Yamanaka, 2006; Eiraku et al., 2011; Canto-Soler et al., 2016; Mandai et al., 2017; Achberger et al., 2019a
Organotypic retinal explants		All retinal cells	Pharmacological research; immunohistochemistry; long-term culture (≤ 4 weeks); technologies: patch-clamp recording, multi-electrode-array recording.	Caffe et al., 1989; Moritoh et al., 2010; Reinhard et al., 2014; Belhadj et al., 2020
Whole eye culture		All retinal cells	Pharmacological research; modeling of ischemic- and perfused- study, pharmacology of vascular dynamics, acute retinal toxicity; technologies: ERG; short-time culture (< 10 h).	Lele and Grimes, 1960; Niemeyer, 1975; Niemeyer, 2001; Koeberle et al., 2006

*The table relates the various in vitro systems to the retinal cell type(s) they may represent, their possible applications in research, and gives select references for each of these systems.*

Notably, cell culture-based systems may be useful to study the intrinsic properties of proteins, as well as their interaction networks, and to compare these with those of corresponding mutant proteins, provided that the cell culture chosen displays a sufficiently high expression of the proteins in question. More complex systems such as co-cultures, retinal organoids, or organotypic retinal explants should be employed if the research questions demand a retinal systems-oriented approach, concerning interdependencies between cell types. Complex *in vitro* culture systems may also be required when highly differentiated primary retinal tissue is to be studied since immortalized cell culture-based systems may lack retina-specific proteins with respect to quantity and quality. The study of even more complex interactions with other organ systems may eventually require the use of *in vivo* animal models. This is especially true for cases where developmental processes or defects are the focus of a study or where the intact eye has to be considered once translational therapy-oriented studies are being conducted.

Nevertheless, the use of *in vitro* test systems features a variety of advantages, including easier and faster molecular and genetic manipulation, reduced experimentation time, and reduced costs compared to animal experimentation. Moreover, hiPSC and their derivatives enable the study of molecular disease mechanisms and the development of therapies in a human and patient-specific context. Last but not least, *in vitro* test systems limit the number of animals used in experiments and replace animal experiments with *in vitro* alternatives, such that live animal research may only be needed in the final stages of pre-clinical development. In this way, clever combinations of *in vitro* systems with limited *in vivo* testing may significantly contribute to advance the implementation of the 3R principles, i.e., the replacement, the reduction, and the refinement of live animal testing for retinal research (Russell and Burch, 1959).

## Author Contributions

AT, BA-G, BC, FP-D, GC, JY, MU, and YZ: writing—review and editing. All authors contributed to one or more chapters of the manuscript, read, and agreed to the published version of the manuscript.

## Conflict of Interest

The authors declare that the research was conducted in the absence of any commercial or financial relationships that could be construed as a potential conflict of interest.

## Publisher’s Note

All claims expressed in this article are solely those of the authors and do not necessarily represent those of their affiliated organizations, or those of the publisher, the editors and the reviewers. Any product that may be evaluated in this article, or claim that may be made by its manufacturer, is not guaranteed or endorsed by the publisher.

## Footnotes

1
http://www.hgmd.cf.ac.uk

## Abbreviations

IRD: inherited retinal degeneration
cGMP: cyclic guanosine monophosphate
PARP: poly (ADP-ribose) polymerase
HDAC: histone deacetylases
RP: retinitis pigmentosa
ONL: outer nuclear layer
INL: inner nuclear layer
RPE: retinal pigment epithelial
RGC: retinal ganglion cell
MGC: Müller glial cell
CNTF: ciliary neurotrophic factor
iPSC: induced pluripotent stem cells
PKG: protein kinase G
DDS: drug delivery system.
